# dRNASb: a systems biology approach to decipher dynamics of host–pathogen interactions using temporal dual RNA-seq data

**DOI:** 10.1099/mgen.0.000862

**Published:** 2022-09-22

**Authors:** Mojdeh Dinarvand, Forrest C. Koch, Daniel Al Mouiee, Kaylee Vuong, Abhishek Vijayan, Afia Fariha Tanzim, A. K. M. Azad, Anahit Penesyan, Natalia Castaño-Rodríguez, Fatemeh Vafaee

**Affiliations:** ^1^​ School of Biotechnology and Biomolecular Sciences, University of New South Wales, Sydney, NSW, Australia; ^2^​ Ingham Institute for Applied Medical Research, Liverpool, NSW, Australia; ^3^​ UNSW Data Science Hub, University of New South Wales, Sydney, NSW, Australia; ^4^​ ProCan®, Children’s Medical Research Institute, Faculty of Medicine and Health, The University of Sydney, Westmead, NSW, Australia; ^5^​ School of Natural Sciences, Faculty of Science and Engineering, Macquarie University, Sydney, NSW, Australia

**Keywords:** dual RNA-seq, host–pathogen interactions, systems biology, bioinformatics tool

## Abstract

Infection triggers a dynamic cascade of reciprocal events between host and pathogen wherein the host activates complex mechanisms to recognise and kill pathogens while the pathogen often adjusts its virulence and fitness to avoid eradication by the host. The interaction between the pathogen and the host results in large-scale changes in gene expression in both organisms. Dual RNA-seq, the simultaneous detection of host and pathogen transcripts, has become a leading approach to unravelling complex molecular interactions between the host and the pathogen and is particularly informative for intracellular organisms. The amount of *in vitro* and *in vivo* dual RNA-seq data is rapidly growing, which demands computational pipelines to effectively analyse such data. In particular, holistic, systems-level, and temporal analyses of dual RNA-seq data are essential to enable further insights into the host–pathogen transcriptional dynamics and potential interactions. Here, we developed an integrative network-driven bioinformatics pipeline, *dRNASb*, a systems biology-based computational pipeline to analyse temporal transcriptional clusters, incorporate molecular interaction networks (e.g. protein-protein interactions), identify topologically and functionally key transcripts in host and pathogen, and associate host and pathogen temporal transcriptome to decipher potential between-species interactions. The pipeline is applicable to various dual RNA-seq data from different species and experimental conditions. As a case study, we applied dRNASb to analyse temporal dual RNA-seq data of *Salmonella *-infected human cells, which enabled us to uncover genes contributing to the infection process and their potential functions and to identify putative associations between host and pathogen genes during infection. Overall, dRNASb has the potential to identify key genes involved in bacterial growth or host defence mechanisms for future uses as therapeutic targets.

## Data Summary

The data set used to showcase the pipeline is available in NCBI’s Gene Expression Omnibus under accession number GSE60144. The pipeline code is available at the GitHub repository https://github.com/VafaeeLab/dRNASb. The authors confirm all supporting data and code have been provided within the article and the GitHub repository or through supplementary data.

**Impact Statement**

Dual RNA sequencing is an important emerging technique to study large-scale transcriptional changes in host and pathogen simultaneously during infection. However, computational tools for the downstream analyses of such data, past the initial read mapping steps, particularly at the systems level are still lacking. This study is aimed to address this methodological need. Here, we describe the development and application of a bioinformatics framework for the analysis of temporal dual RNA-seq profiles of pathogens and their host cells during intracellular infection. We established a general, network-driven pipeline for the analyses of such data with wide applicability and impact on the microbial genomics community.

## Introduction

Infectious diseases continue to be a major threat worldwide, with devastating impacts on lives and economies around the globe. A better understanding of microbial infection mechanisms is central to the development of new antimicrobial therapies [1]. During bacterial infections, a complex interplay is engaged by both bacterium and the host to negotiate the respective survival and defence strategies [2]. Unravelling pathogen and host regulatory interactions, virulence mechanisms and innate responses has led to the understanding of the dynamics of infectious processes and the development of therapeutics [3, 4]. In studying infections, analysing the transcriptomes of both organisms involved is key to better understand pathogenesis and disease progression [5, 6]. Dual RNA sequencing (dual RNA-seq) is an emerging powerful tool to capture whole transcriptomes, including coding and non-coding RNAs, of both host and pathogen simultaneously in order to dissect the host-pathogen interplay and reveal the impacts that both organisms exert over each other during infection [4, 7–9]. Dual RNA-seq experiments are often designed to capture transcriptomics profiles of both organisms at different time-points after infection to enable the study of the molecular dynamics underlying host response and bacterial fitness [10]. To understand the strategies employed by a pathogen for controlling or progressing infection, a growing number of dual RNA-seq experiments have been conducted under *in vivo* and *in vitro* conditions [2–6] for pathogens such as *Yersinia pseudotuberculosis* [11], *Pseudomonas aeruginosa* [12, 13]*,* and *Mycobacterium leprae* [14], as well as intracellular bacteria such as *Mycobacterium tuberculosis* [7, 15] and *Piscirickettsia salmonis* [16].

Dual RNA-seq experiments inherently generate a mixture of transcripts from both host and pathogen, requiring computational approaches to sort this mixture into the components associated with each organism. Multiple analytical workflows have been developed to align this read mixture to the reference genomes of the host and pathogen in model organisms [4, 7, 17] as well as non-model systems by leveraging the genomic resources of closely-related species [18]. The majority of dual RNA-seq bioinformatics pipelines generated to date have been focused on upstream analyses (e.g. quality control, alignment, read filtration, and normalisation) [18], with downstream analyses often limited to differential expression and functional analyses [7, 17]. Hence, there is a need for computational resources that offer more holistic and temporal downstream analyses of host and pathogen transcriptome, enabling further insights into the host-pathogen transcriptional dynamics and potential interactions. In particular, systems biology approaches which integrate information on molecular interactions with gene expression information [19, 20], can provide a more holistic understanding of molecular intricacies underlying the infection process. However, the application of systems biology is still limited in this context.

To address this resource need, we developed an integrative network-driven bioinformatics pipeline, *dRNASb* (dual RNA-seq *Systems biology-based analysis*), to analyse temporal transcriptional clusters, incorporate molecular interaction networks (e.g. protein–protein interactions), identify topologically and functionally key transcripts in host and pathogen, and associate host and pathogen temporal transcriptome to decipher potential between-species interactions. The pipeline is applicable to various dual RNA-seq data from different species and experimental conditions. As a case study, we applied dRNASb to analyse dual RNA-seq data of *Salmonella *-infected human cells [21] at six time-points post-infection (pi), which enabled us to uncover genes contributing to *Salmonella* infection and to identify their potential functions.

## Methods

### Data pre-processing and differential gene expression analysis

The pipeline accepts a dual RNA-seq tab-delimited input file where read counts from both the host and the bacterium are collapsed into a single matrix of genes (rows) and time-points (columns). Rows with more than 50 % missing values were filtered out. Raw counts were normalised using the trimmed mean of M-values (TMM), which corrects for differences in RNA composition and sample outliers while providing better comparability across samples. Normalised data were then log_2_ transformed. Before differential expression analysis, reads were separated to the pathogen and host genome using the respective reference genomes (provided as input). Then, the *limma* package in R was used to identify differentially expressed (DE) genes, which uses linear modelling to describe expression data [22]. The output is a list of DE genes compared with the baseline (i.e. 0 h). Genes with a false discovery rate (FDR) cut-off ≤0.05 (i.e. 5 % false positives) and fold-change of at least two-fold in either direction (upregulation/downregulation), i.e. log2(fold-change)|>1, were considered as statistically significant DE genes. These lists can then be used as an input for the analysis of temporal gene expression profiles.

### Temporal analyses of gene expression profiles

To unravel transcriptional dynamics, fuzzy clustering was conducted on DE genes of at least one time-point. We used the Mfuzz package in R [23], which is developed for noise-robust soft clustering of gene expression time-series data. The number of clusters should be first identified for which we performed repeated soft clustering for a range of cluster numbers using ‘*Dmin*’ function and reported the minimum centroid distance (MCD), i.e. the minimum distance between two cluster centres. The optimal cluster number was then determined by plotting MCD against cluster numbers and selecting a number where there is a drop of MCD and a slower decrease afterwards (a.k.a. the elbow rule). After soft clustering, each gene is assigned a membership score representing the degree of its association to each cluster that reflects a particular temporal pattern. The objective of Mfuzz is to compute membership values to minimise the within-cluster distances and maximise the between-cluster distances. The output of Mfuzz is a membership list for each gene class. The membership value ranges from 0 to 1, with higher values indicating that a gene is more likely to belong to a particular class.

### Pathway enrichment analyses for host and pathogen

To identify potential functions associated with DE genes in each temporal cluster, the Cluster Evaluation R (clueR) package in R [24] was used to assess the enrichment of different functional terms by DE genes in each cluster, using Fisher’s exact test with hypergeometric null distribution to calculate the probability of pathways associated with a set of DE genes within a cluster; adjusted *P*<0.05 was considered to indicate statistically significant associations. Enrichment analysis relies on a reference dataset that annotates genes based on associated pathways or other functional terms. A database of pathways, regulons, and genomic islands was constructed using information obtained from the BioCyc.org Database for the bacterium (i.e. *Salmonella enterica SL1344*). BioCyc.org is unique in integrating a diverse range of data and providing a high level of curation for important microbes as well as model eukaryotic organisms and *Homo sapiens* [25]. The Kyoto Encyclopaedia of Genes and Genomes (KEGG) and Gene Ontology Consortium (Amigo: http://amigo.geneontology.org) were used to retrieve functional annotations for the host (i.e. human).

### Gene ontology enrichment analysis and visualisation for host and pathogen

Gene Ontology (GO) is an annotation database structured as a hierarchical directed acyclic graph where each node represents a class of gene function (GO term), and the connection between two GO terms indicates different relationships, such as ‘is a’ or ‘part of’. GO terms are categorised into biological processes (BPs), molecular functions (MFs) and cellular components (CCs) [26]. Statistical enrichment of GO terms was conducted using the Enrichr tool which comprise an up-to-date libraries of diverse gene sets including gene ontology BP, MF and CC gene sets [27]. Similarly, statistical overrepresentation analyses were performed using Fisher’s exact test with the hypergeometric null distribution. Visualisation of enrichment was accomplished via bar plots and bubble plots using R scripts.

### Protein-protein interaction network construction, analyses, and visualisation

A protein–protein interaction (PPI) databases for the pathogen (e.g. SalmoNet, salmonet.org [28] for the pathogen of interest in this study) and host (e.g. human STRING, string-db.org [29]) were retrieved, and the corresponding network was constructed using the *igraph* package [30] for network topology analyses. The STRING database can be used to retrieve PPIs for several other host and/or pathogen species of interest. Its current version (v11) covers functional and physical interactions among 24.6 million proteins from 5 090 organisms [31, 32]. Multiple network measures, including node degrees, betweenness centrality [33], closeness centrality [33] and modularity (via community detection) were identified. The functions of the hub genes (i.e. those genes with a high degree of connectivity) were identified as described before. Community structure detection was constructed using the cluster edge betweenness function in the *igraph* package to cluster similar groups of hub genes (i.e. those which have the highest similarity degree) [34].

### Inter and intraspecies correlation analysis of pathogen and host transcriptome

Pearson correlation coefficients were calculated between and across host and pathogen DE genes, and *P* values were calculated using the function *cor.test* in R. To account for a possible temporal delay between *Salmonella* expression changes and effect manifestation in the host cell, a time-shift was allowed. This means the expressions of pathogen genes at each time-point were compared to the host expression at the subsequent time point. Once the correlation network was built (correlation coefficient <−0.7 or >0.7, and adjusted *P*<0.05), densely connected genes were identified via Louvain clustering, which can quickly find clusters with high modularity in large networks [35]. Host and pathogen genes within each cluster were further investigated to identify genes potentially important during the early stage of infection.

### Pipeline access and usage notes

The pipeline code is available at GitHub repository https://github.com/VafaeeLab/dRNASb. The pipeline is reusable and applicable to different dual RNA-seq datasets. Users need to provide related input files, including a *phenotype file* (specifying samples and groups/conditions), *expression data* (read counts of transcripts in host and pathogen), *functional annotations,* and *PPIs* of the species of interest. The repository also provides example input files as well as self-contained documentation describing the pipeline, scripts, and folders. The package is available as a library that can be installed on the end-user machines as per the instructions available.

## Results and discussion

**Fig. 1.**
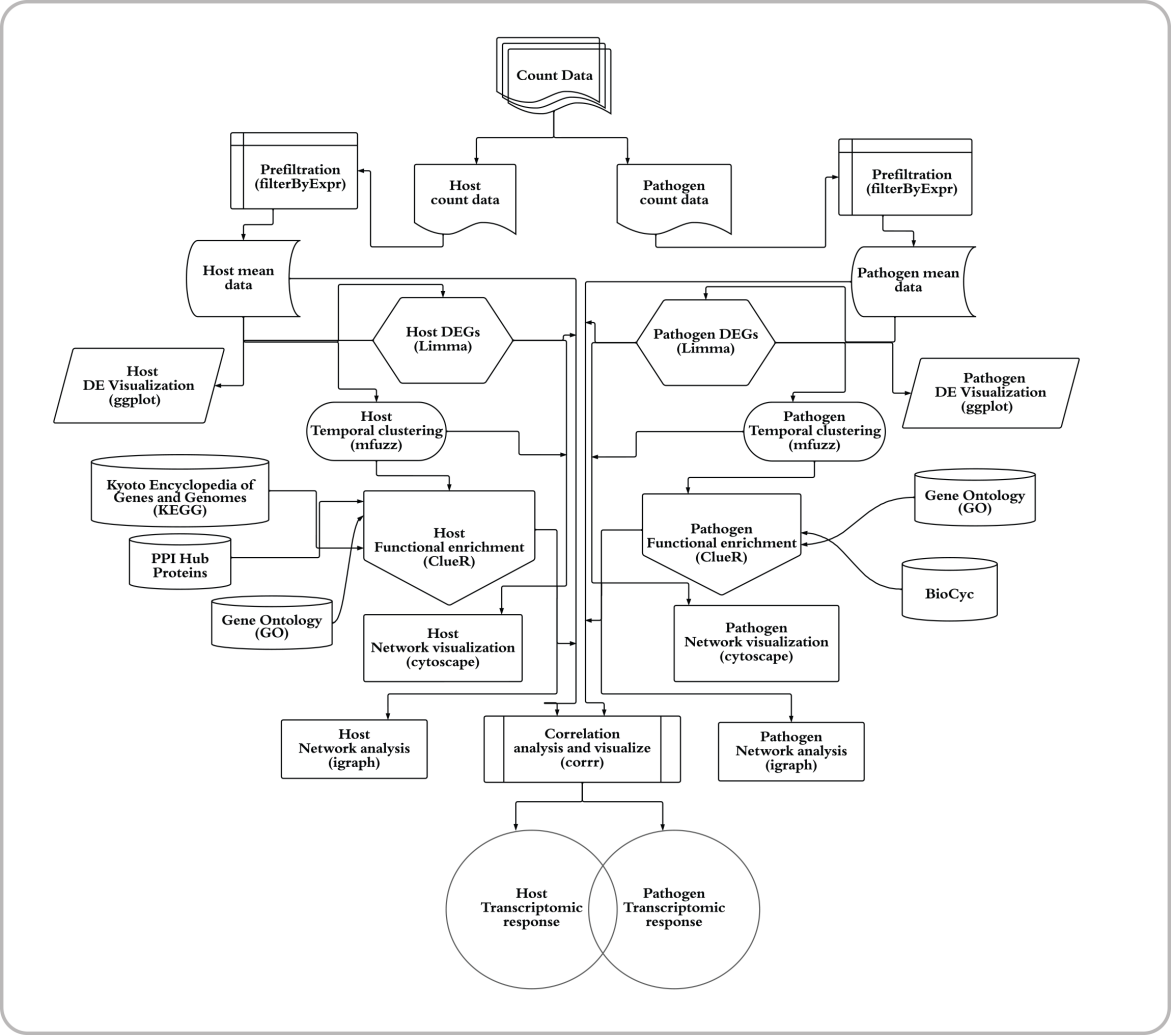
Flowchart of the bioinformatic pipeline for the dual RNA-seq analysis of a host–pathogen interaction. The pipeline starts with data pre-processing and differential gene expression analysis following by Fuzzy clustering to decipher coherent patterns of temporal gene expression profiles. The pathway enrichment analysis was applied using KEGG and GO annotations to identify functions overrepresented by temporal clusters in host and pathogen (based on Fisher’s exact test with hypergeometric null hypothesis). To explore relationship and potential physical or regulatory interactions among differentially expressed genes, the protein–protein interaction (PPI) for both species and regulatory networks for pathogens were retrieved from different datasets. The topological characteristics of the genes were then identified. Additionally, the gene co-expression networks were constructed to infer cross-species gene associations and then used to identify hubs, betweenness centrality, closeness centrality and modularity followed by functional analysis.

The dRNASb bioinformatics pipeline comprises the following steps: filtering and normalisation of count data, identification of DE genes in the host and pathogen, temporal gene expression clustering, functional enrichment analyses, network analysis, visualisation, and inter/intraspecies correlation analyses (Fig. 1). The pipeline, as mentioned above, is coded in R (version-4.0). The model system used in this work to study the bioinformatic pipeline comprised human cervix carcinoma cells (HeLa-S3; ATCC CCL-2.2) (host) infected with *Salmonella enterica* serovar Typhimurium strain SL1344 (bacterium), which is a well-defined host–bacteria system [36]. The pipeline, however, can be repurposed to analyse temporal dual RNA-seq data from other host-bacteria systems of interest.

**Fig. 2.**
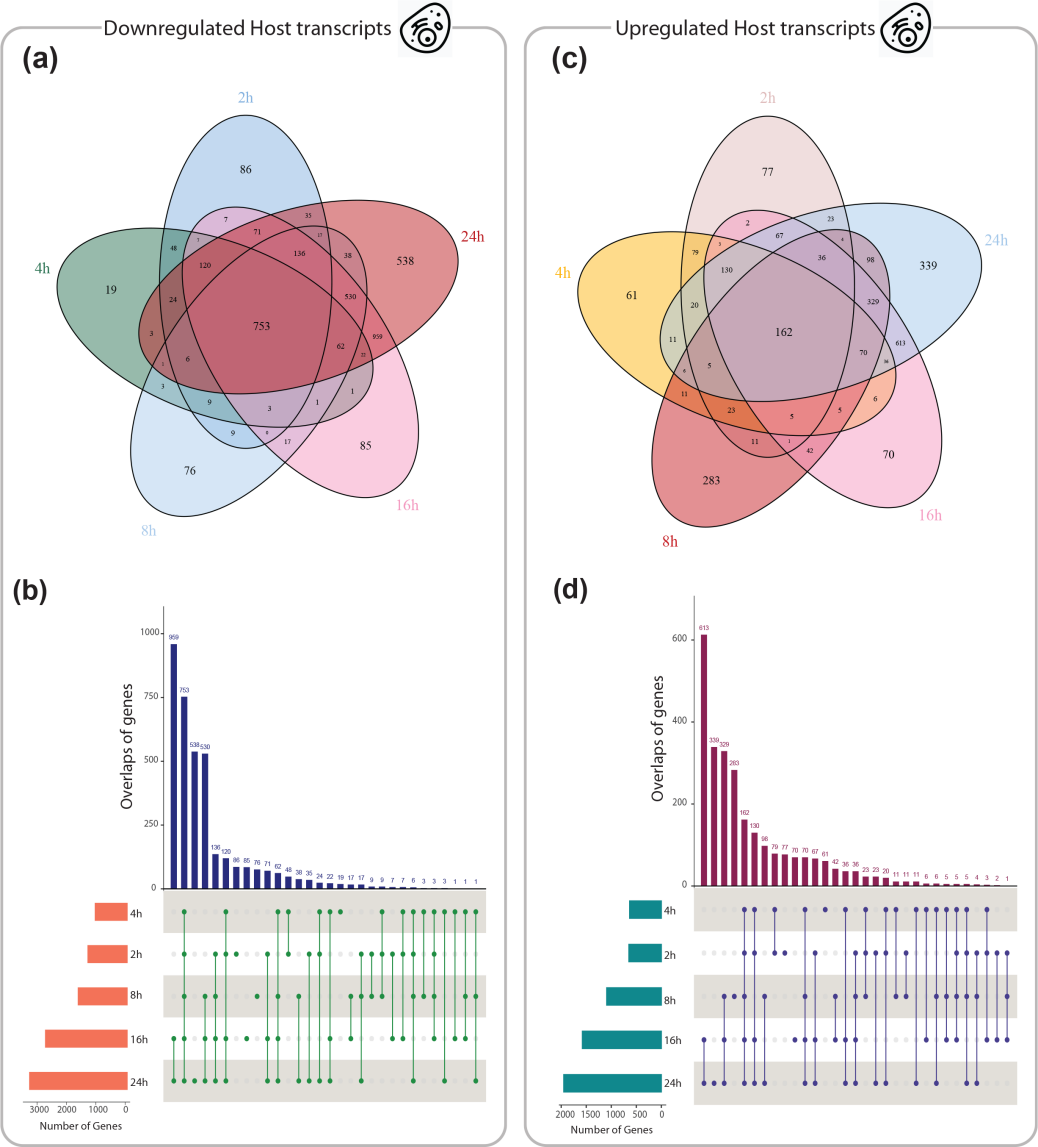
Visualisations of overlaps among *host* genes upregulated (3 686 genes) or downregulated (2 628 genes) across five different time points post-infection compared to baseline (0 h) (adjusted *P*<0.05, |log2(fold-change)|>1) using Venn diagrams (**a and c**) and UpSet plots (**b and d**). While Venn diagrams are visually more familiar, UpSet plots [86] are better suited for the quantitative analysis of data with more than three sets by visualising set intersections in a matrix layout.

**Fig. 3.**
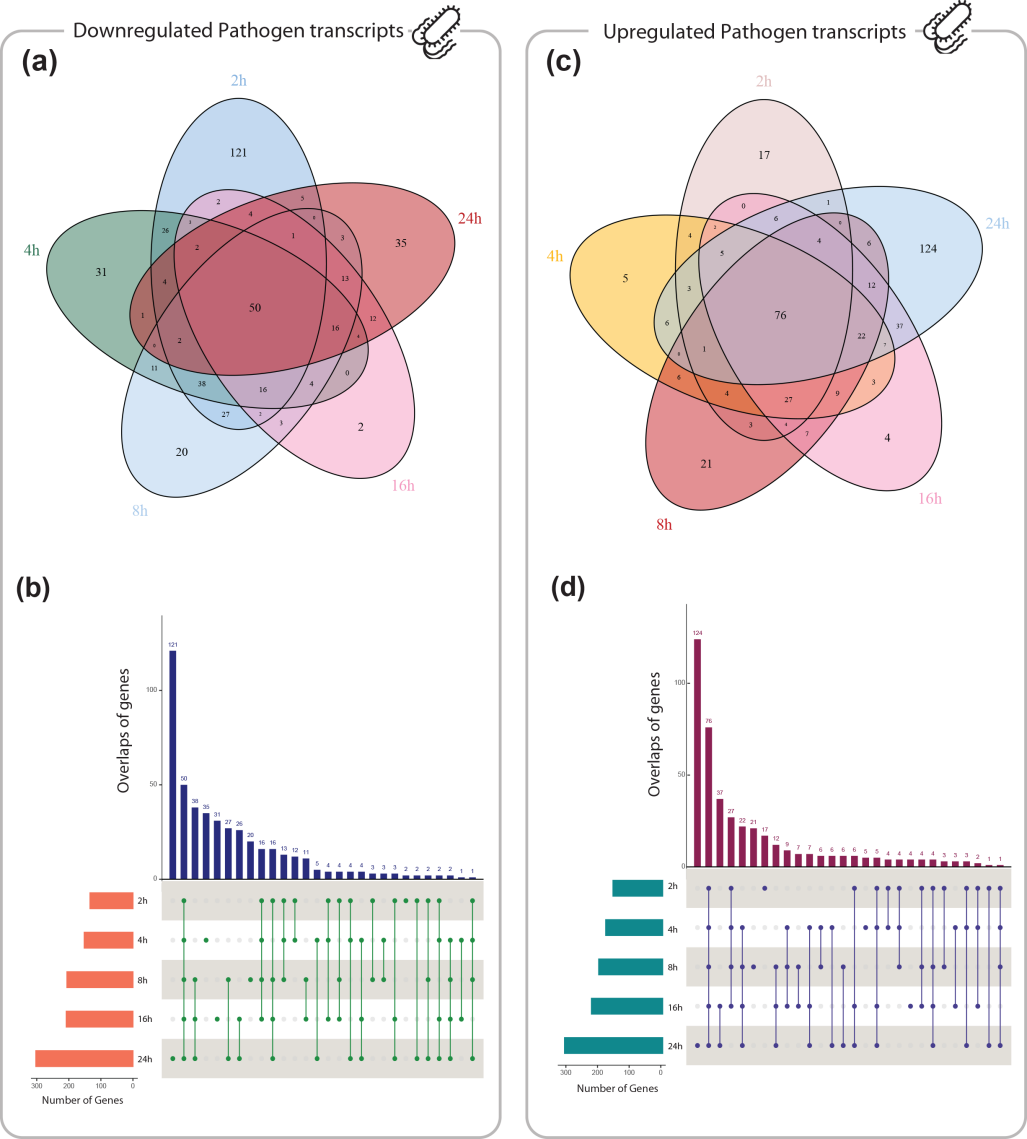
Visualisations of overlaps among *pathogen* genes upregulated (426 genes) or downregulated (458 genes) across five different time points post-infection compared to baseline (0 h) (adjusted *P*<0.05, |log2(fold-change)|>1) using Venn diagrams (**a and c**) and UpSet plots (**b and d**).

The dual RNA-seq read count matrices were downloaded from Gene Expression Omnibus (accession number GSE60144) [21], quantifying the expression level of host and pathogen transcripts across six time-points (0, 2, 4, 8, 16, and 24 h). Details of upstream analyses including read processing and mapping are available in the original paper [25]. Gene expression levels in the host and pathogen at different times compared to the start of the experiment (0 h) were evaluated, and DE genes (adjusted *P-*value <0.05 and |log2(fold-change)|>1) were identified. In total, 6 248 and 831 DE genes were found in the host and pathogen, respectively, that were differentially expressed at least in one time-point Table S1. The overlap of upregulated and downregulated DE genes in the host and pathogen is displayed in Figs 2 and 3, respectively. As can be seen, the number of downregulated genes in the host (Fig. 2a) and the pathogen (Fig. 3a) was significantly increased at 2 h and 24 h pi (compared to pre-infection), respectively. A higher number of upregulated genes in both organisms was observed at 24 h pi. Venn diagrams in Figs 2 and 3 also present the number of genes dysregulated specific to each time point.

In general, genes with similar expression patterns which form temporally co-expressed clusters have the potential to exhibit similar cellular functions as guided by the ‘guilt-by-association’ assumption [37]. Accordingly, fuzzy clustering algorithm, which produces overlapping clusters was applied to the 6 248 host and 831 pathogen genes with expression time series of length 6. The optimal number of clusters was ten and determined as previously explained (see Methods). The membership degree was set to ≥ 0.5 to select genes that tightly follow the average expressional pattern of each cluster and remove genes which do not strongly belong to any cluster.

**Fig. 4.**
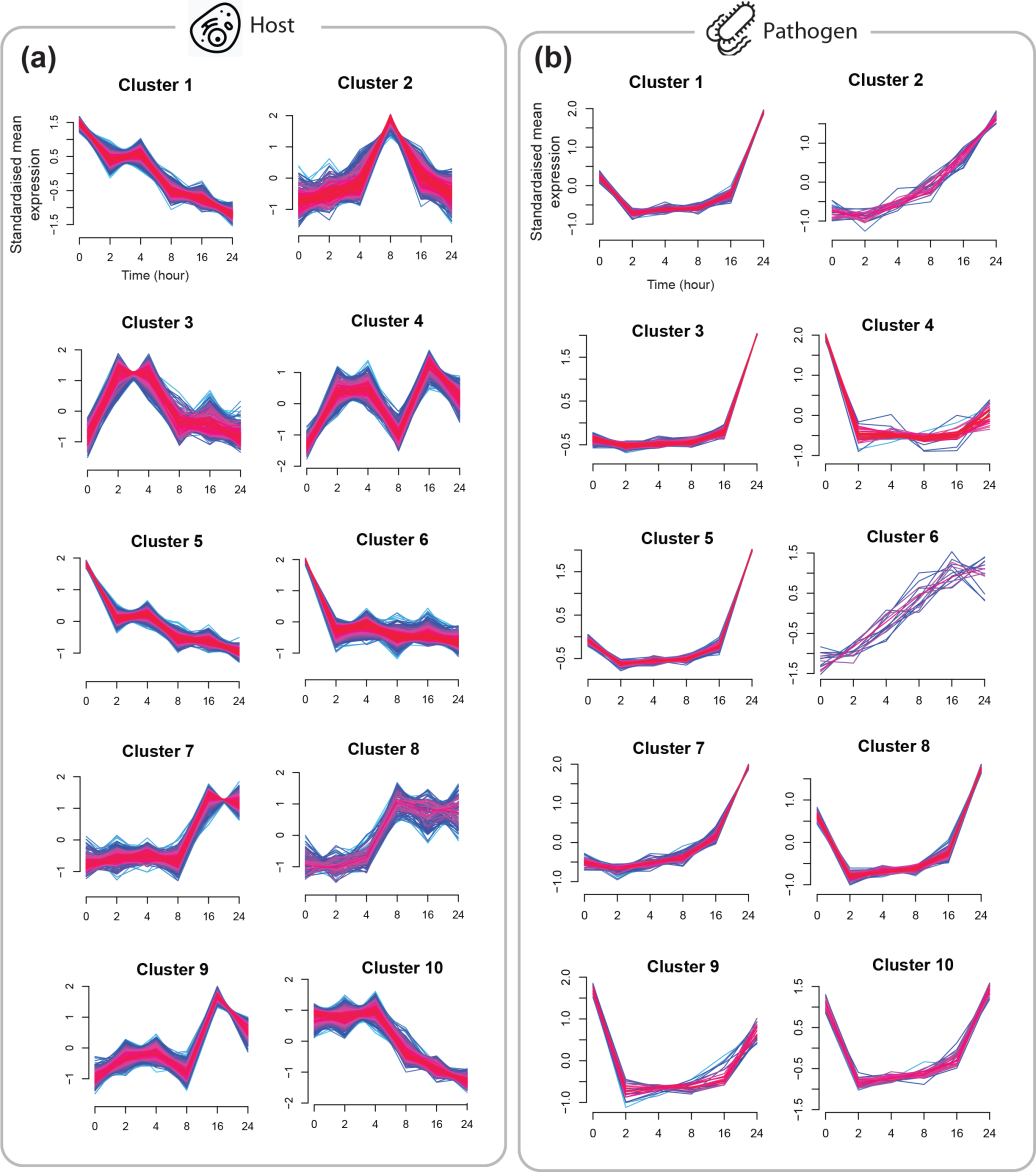
Line plots representing temporal gene-expression profiles of host (**a**) and pathogen (**b**) DE genes clustered based on the similarity of their temporal patterns using fuzzy clustering. The membership degree was set to ≥0.5 to select genes that tightly follow the average expressional pattern of each cluster and remove genes which do not strongly belong to any cluster. The y axis shows the z-standardised expression values.

Accordingly, 4 893 out of 6 248 genes grouped into ten clusters (Fig. 4a) and the most populated expression pattern observed as upregulation and downregulation activities peak at 24 h (pi) in clusters six and ten, respectively (Table 1). On the other hand, the result of pathogen clustering showed 690 out of 831 pathogen genes were grouped into ten clusters (Fig. 4b); the most populated expression pattern observed as an up-regulation and down-regulation activities peak at 24 h (pi) in clusters three and ten, respectively (Table 2).

**Table 1.** Summary of DEGs distribution in the host clusters

Timepoint (pi)	Total genes	Upregulated	Downregulated
2h	1 646	492	1 154
4h	1413	449	964
8h	2 164	702	1 462
16 h	3 620	1 151	2 469
24 h	4 328	1 412	2 916

**Table 2.** Summary of DEGs distribution in the pathogen clusters

Timepoint (pi)	Total genes	Upregulated	Downregulated
2h	381	115	122
4h	324	137	187
8h	328	146	182
16 h	310	190	120
24 h	408	286	266

The functional annotation and enrichment analysis on clusters of DE genes using GO terms and KEGG allowed us to obtain functions enriched by temporal clusters of DE genes in host (Table S3) and pathogen (Table S2) during the infection process.

Overall, 20 genes from four pathogen clusters were enriched with two main classes of cellular functions important for pathogenesis (Table S2). The first class was related to the transmembrane transporter activity, electron transport chain, and the biosynthesis of a micronutrient such as biotin that acts as a co-factor for bacterial metabolic activities. The role of biotin biosynthesis and transport functions in bacterial pathogens have been well studied [38–40]. Therefore, understanding the transcriptional mechanisms regulating essential elements of biosynthesis and transport will extend our knowledge on bacterial survival and metabolic adaptation during pathogenesis.

The peptidoglycan biosynthetic pathway is a critical process in the bacterial cell [41] and the interplay between peptidoglycan biology and pathogenesis has been previously reported [42]. The enrichment analysis has shown that the second class of functions overrepresented by pathogen DE clusters (i.e. cluster 8, genes *tig* (E1W8V7) and *ftsK* (E1W6Z2)) included the regulation of cell shape and peptidoglycan synthesis that enable bacteria to maintain, modify, and reshape the peptidoglycan layer without risking its essential functions in cell shape and cellular integrity. DE analysis shows that these genes are downregulated during the first 8 h and upregulated afterwards. Peptidoglycan is generally considered one of the main determinants of the cell shape maintenance in bacteria [43]. The outcome of this study supports this function: during earlier hours of infection, expressions of these genes were downregulated, which may indicate that the bacteria’s shape is changing. After the bacterial invasion of host cells, the expression of these genes was sharply upregulated, which can be the result of duplication of bacteria and the initiation of infection.

During the initial steps of infection, pathogens manipulate and modify host cell biology to ensure conditions best suited for colonisation [44]. This process can stimulate the response of the innate immune system [45]. GO enrichment analysis has shown that 380 genes across host clusters were enriched with functions that play essential roles in mediating cellular entry of pathogens (e.g*.* epidermal growth factor receptor (EGFR) signalling pathway [46], growth hormone receptor signalling pathway [47], mitochondrial ATP synthesis coupled electron transport [48], protein targeting to the endoplasmic reticulum [49]), communication of bacteria with the host environment (e.g*.* positive regulation of establishment of protein localisation [50]), control of infection (e.g*.* regulation of protein metabolic process [51]), invading pathogen (e.g*.* response to laminar fluid shear stress [52]), and co-opting host factors (e.g*.* viral gene expression [53]) that can be considered as potential targets in designing antibacterial therapies. Enriched functions were mostly observed at 24 h and 4 h pi in the host and pathogen, respectively, with mainly downregulated expression.

Integration of biological networks, such as protein–protein interactions, has shown to be a useful approach to correlating gene expression changes with changing conditions [54, 55] and can provide information about the most significant connections among classified DE genes in the host and pathogen during early stages of infection. We, therefore, overlaid DE genes across different clusters on the protein–protein interaction (PPI) network of the host and pathogen to show how genes and their products of different clusters can possibly cross talk. We also constructed PPI networks among *all* DE genes (not exclusive to those genes that appeared in temporal clusters) in the host and pathogen that, in this study, are referred to as the host gene network (HGN) and the pathogen gene network (PGN), respectively.

**Fig. 5.**
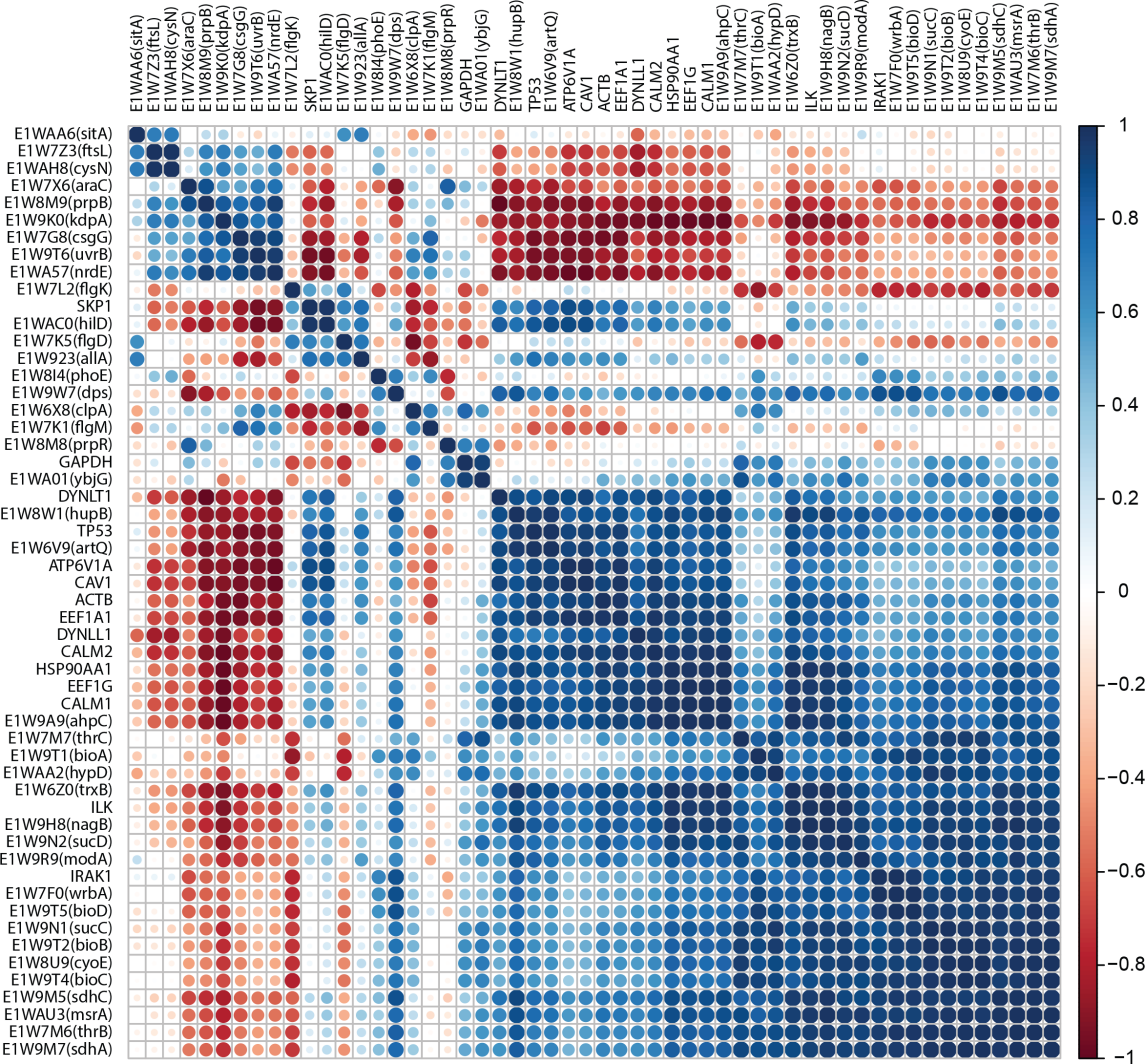
Correlation analysis has shown that hub genes, i.e. nodes with degree >10 for pathogen and >100 for host, separately identified in the host and pathogen co-expression networks (129 and 293 hubs in host networks, respectively), are significantly correlated. Network community detection (i.e. Louvain clustering algorithm) applied on hub genes (i.e. degree >10 for pathogen and >100 for host) with high betweenness (≥40) resulted in four inter-connected modules in negatively correlated (corr <−0.7) network (**a**) and three modules in positively correlated (corr >0.7) network (**b**).

In order to detect key genes and clusters of genes with functionally enriched pathways during early stages of infection, a gene co-expression network analysis using *igraph* package was performed. Co-expression modules identified by clustering are often large, and therefore, it is important to identify key gene(s) in each module. A widely used approach is to identify highly connected genes in a co-expression network (i.e. hub genes). A hub gene is a gene associated with many other genes in gene networks. Additionally, centrality measures, mainly ‘betweenness centrality’, are often used to identify topologically important genes in a biological network [56]. High betweenness centrality of a gene indicates that the node/gene serves as a connector in the network and lies in many shortest paths that connect together different parts of the network [57]. Genes with high degree and betweenness centrality may play a key role in biological processes and gene regulation [58] and tend to be highly relevant to the functionality of biological networks. Many biological networks are organised into connected substructures or modules which carry particular functions within the system [59]. Hub genes can be identified within a module (rather than the entire network), that is referred to as intra-modular hubs and can be central to a specific function carried by the module. Correlation analysis has shown that hub genes, i.e. nodes with degree >10 for pathogen and >100 for host, separately identified in the host and pathogen co-expression networks (129 and 293 hubs in host networks, respectively), are significantly correlated. We also applied network community detection (Louvain clustering algorithm) on hub genes and identified four inter-connected modules in negatively correlated (corr <−0.7) (Fig. 5a and Tables S4–S7) network and three modules in positively correlated (corr >0.7) (Fig. 5b and Table S8–S10) network. These modules can potentially be involved in the same or similar biological processes.

To identify potential cellular functions and diseases associated with genes contained in each module, we performed KEGG pathway, KEGG disease, and GO enrichment analyses on the set of genes contained in each of the negatively and positively correlated modules. Analyses have shown that the host and pathogen gene sets in the negatively and positively correlated modules were enriched with similar as well as host-/pathogen-specific functions (Table 3). For instance, the most frequently enriched functions specific to the host gene set in the first negatively-correlated module were involved in processes underlying the protection against infectious diseases, and the innate immune system which employs germline-encoded pattern-recognition receptors (PRRs) and Toll-like receptors [60–62]. These distinct processes are activated upon detection of the initial natural infection mediated by a pathogen or immunisation.

**Table 3.** Enrichment analysis (KEGG pathways and Gene Ontology) by host and pathogen DE genes in each of positively- and negatively-correlated modules

Negatively-correlated Modules				
Module No.	Pathogen gene set	Functions enriched by pathogen gene set	Host gene set	Functions enriched by host gene set
1	E1W6Z0 (*trxB*), E1W7A3 (*sulA*), E1W7G8 (*csgG*), E1W7G8 (*csgG)*, E1W7×6 (*araC*), E1W7Y7 (*leuO*), E1W7Z3 (*ftsL*), E1W8M8 (*prpR*), E1W8M9 (*prpB*), E1W9K0 (*kdpA*), E1W9R9 (*modA*), E1W9T6 (*uvrB*), E1WAA6 (*sitA*), E1WAR7 (*ulaG*)	membrane and metal ion transport activity	*IKBKG, VCP*	regulation of cytokine-mediated, stimulatory C-type lectin receptor, toll-like receptor, cytoplasmic pattern recognition receptor and JNK
2	E1W6×8 (*clpA*), E1W7F0 (*wrbA*), E1W7M7 (*thrC*), E1W8I4 (*phoE*), E1W923 (*allA*), E1W9H8 (*nagB*), E1W9T1 (*bioA*), E1W9W7 (*dps*), E1WA01 (*ybjG*), E1WA57 (*nrdE*), E1WAA2 (*hypD*), E1WAH8 (*cysN*)	metabolic process, nucleotidyl transferase activity, and ion transmembrane transport	*HSP90AA1*, *SQSTM1*	vascular endothelial growth factor receptor, ERBB2 pathway, and Fc receptor mediated stimulatory, mitochondrial transport, G2/M transition of mitotic cell cycle, regulation of cellular protein metabolic process, protein transmembrane import into intracellular organelle, regulation of transferase activity, autophagy of mitochondrion, regulation of cellular response to stress and heat, regulation of mitochondrion organisation, regulation of apoptotic process, and programmed cell death
3	E1W7M6 (*thrB*), E1W9M5 (*sdhC*), E1W9M7 (*sdhA*), E1W9T2 (*bioB*), E1W9T4 (*bioC*), E1W9T5 (*bioD*), E1WAU3 (*msrA*)	transferase activity, biotin biosynthetic process, metal ion binding, iron-sulphur cluster binding, and oxidation-reduction process	*EEF1A1, EEF1G, GAPDH, ILK, IRAK1, MYC, SKP1, TP53, ACTB, ATP6V1A, CALM1, CALM2, CAV1, DYNLL1, DYNLT1*	G1/S transition of mitotic cell cycle, neutrophil activation involved in immune response, translation elongation factor activity, GTPase activity, lysosomal membrane, regulation (autophagy, endopeptidase activity, microtubule cytoskeleton, macroautophagy, protein metabolic process, cellular amide metabolic process, chaperone-mediated autophagy, ficolin-1-rich granule lumen, glutathione metabolic process, cellular macromolecule biosynthetic process, cadherin binding, signal transduction, myeloid leucocyte differentiation, iron ion homeostasis) and response (interferon-gamma, antimicrobial humoral immune response mediated by antimicrobial peptide, interferon-gamma, disordered domain specific binding)
4	E1W6V9 (*artQ*), E1W7K1 (*flgM*), E1W7K5 (*flgD*), E1W7L2 (*flgK*), E1W8U9 (*cyoE*), E1W8V2 (*cyoB*), E1W8W1 (*hupB*), E1W9A9 (*ahpC*), E1W9N1 (*sucC*), E1W9N2 (*sucD*), E1WAC0 (*hilD*)	bacterial-type flagellum organization/hook/assembly, integral component of membrane, regulation of cellular macromolecule biosynthetic process, ligase activity and aerobic respiration	*NFKB1*	This is a master transcription factor involved in many processes including inflammation, immunity, differentiation, cell growth, tumorigenesis, and apoptosis. However, an individual gene cannot statistically enrich any function.
**Positively-correlated Modules**				
1	E1W8U9 (*cyoE*), E1W8W1 (*hupB*), E1W9A9 (*ahpC*), and E1WAC0 (*hilD*)	integral component of membrane, transferase activity, response to oxidative stress, and regulation of cellular macromolecule biosynthetic process	*GAPDH* and *UBC*	response (stress, intracellular transport of pathogen, cellular protein metabolic process, fibroblast growth factor stimulus), regulation (mRNA stability, epidermal growth factor receptor, transmembrane receptor protein serine/threonine kinase, establishment of planar polarity, intracellular signal transduction), modification of dependent macromolecule catabolic process, and small protein removal which are key functions during infection
2	E1W6×8 (*clpA*), E1W6Z0 (*trxB*), E1W7F0 (*wrbA*), E1W7G8 (*csgG*), E1W7G8 (*csgG*), E1W7M6 (*thrB*), E1W7M7 (*thrC*), E1W7×6 (*araC*), E1W7Z3 (*ftsL*), E1W8I4 (*phoE*), E1W8M8 (*prpR*), E1W8M9 (*prpB*), E1W923 (*allA*), E1W9H8 (*nagB*), E1W9K0 (*kdpA*), E1W9M5 (*sdhC*), E1W9M5 (*sdhC*), E1W9M7 (*sdhA*), E1W9M7 (*sdhA*), E1W9M7 (*sdhA*), E1W9R9 (*modA*), E1W9T1 (*bioA*), E1W9T2 (*bioB*), E1W9T4 (*bioC*), E1W9T5 (*bioD*), E1W9T6 (*uvrB*), E1W9W7 (*dps*), E1WA01 (*ybjG*), E1WA57 (*nrdE*), E1WAA2 (*hypD*), E1WAA6 (*sitA*), E1WAH8 (*cysN*), E1WAU3 (*msrA*)	protein metabolic process, protein unfolding, cell adhesion, GTP binding, regulation of cellular macromolecule biosynthetic process, integral component of plasma membrane, metal ion transmembrane transport, and electron transport chain	*EGFR, GNAQ, MAPK14, PIK3C2A, PRKCE, PXN, ACTN1*	regulation (cell migration, cell population proliferation and differentiation, intracellular signal transduction, protein localization to plasma membrane, reactive oxygen species metabolic process, interleukin-12 secretion, sprouting angiogenesis, carbohydrate metabolic process, fibroblast migration, ion transmembrane, transporter activity, wound healing, focal cell-matrix and adhesion, actin filament network formation) and response (UV, early endosome membrane, metal ion, lipopolysaccharide, growth factor stimulus, ionizing radiation, pathogen, myoblast fusion oxygen-containing compound, oxidative stress, stress fibre)
3	E1W6V9 (*artQ*), E1W7K1 (*flgM*), E1W7K5 (*flgD*), E1W7L2 (*flgK*), E1W9N1 (*sucC*), E1W9N2 (*sucD*), E1WAB2 (*sirC*)	transmembrane transporter activity, integral component of membrane, bacterial-type flagellum assembly, and organisation	*EEF1A1*, *EEF1G*, *HSP90AA1*, *ILK*, *IRAK1*, *SKP1*, *TP53*, *ACTB*, *ATP6V1A*, *CALM1*, *CALM2*, *CAV1*, *DYNLL1*, *DYNLT1*	vascular endothelial growth factor receptor, cytoplasmic pattern recognition receptor, Fc-gamma receptor, cell surface receptor and leucine rich repeat containing receptor, regulation (entry of bacterium into host cell, cell communication by electrical coupling, autophagy, intracellular signal transduction, reactive oxygen species metabolic process, population proliferation, intracellular pH reduction, transition metal ion homeostasis, cation channel activity, endothelial cell proliferation, receptor internalisation) and response (heat, unfolded protein, stress, lipopolysaccharide, molecule of bacterial, origin ionising radiation, increased oxygen levels, outer mitochondrial membrane organisation, intracellular protein, metal ions transmembrane)

The mitochondria metabolic process was the main function enriched by host genes in the second negatively correlated module. This function has a central role in regulating cellular activities [63] and directly controls host stimulated responses against infections [63]. Yet, multiple pathogens have the ability to manipulate mitochondrial dynamics and functions as well as cell metabolism and immune responses [64, 65].

The Wnt signalling pathway was a dominant pathway enriched by the host gene set in the third negatively-correlated module. This pathway contributes to the cell cycle control, cytoskeleton reorganisation during phagocytosis and cell migration, autophagy, apoptosis, and a number of other inflammation-related events [66]. It has also emerged as an integral component of host responses to infection, but its function in the context of immune responses is not fully understood [66–68]. Recent studies have shown that this pathway can control host processes related to bacterial infection; however, pathogens have evolved strategies to manipulate Wnt-associated processes in order to enhance infection and survival within the human host [66–68]. The mitogen-activated protein kinase (MAPK) signalling pathway was the second most enriched pathway by this set of genes. This pathway plays a central role in host-pathogen complex interactions and is pivotal for triggering the host immune response against pathogens [69, 70].

In the fourth negatively-correlated module, lipid droplets (LDs) were enriched by the host gene set, which are the choke point in the conflict between host and pathogen for nutrients [71, 72]. Previous studies on the nutritional status of the infected host have discussed the malfunctioning or lack of LDs as a key player in the regulation of systemic innate immunity [72]. On the other hand, bacteria can uncouple LDs from mitochondria and increase host–pathogen contacts by reducing fatty acid metabolism [73]. Yet, the nature of the distinct junction between the front line of the body defence (LDs) and bacteria is not well unknown [72]. Furthermore, recent studies have revealed pathogen ability in the manipulation of the host membrane to facilitate pathogenic entry and recruitment of specific host lipids for the maintenance of favourable intracellular niche to augment their survival and proliferation [71, 72, 74].

In the first positively-correlated module, the host genes enriched the Notch signalling pathway that is critically involved in developmental processes and regulating immune responses during infections [75]. Two main signalling pathways enriched by the host gene set in the second positive module included G protein-coupled receptors and epidermal growth factor receptors (EGFR). These are involved in the pathogen pathophysiological processes [76, 77] and in mediating cellular entry of numerous pathogen cells [46], respectively. Generally, the functions related to this module have a role in immune pathogenesis by activities of innate and adaptive immune systems.

Leucine-rich repeats (LRRs) were enriched by the host gene set in the third positively-correlated module. The leucine-rich repeat domain presents in several innate immune receptors, where it is frequently responsible for sensing danger signals and specific pathogen-associated molecules. It will be active the innate immune system as a host defence system, where they sense specific pathogen-associated molecules [78]. Fc-gamma and cell surface receptors were the second signalling pathways which were enriched by the host gene set. These receptors are an essential component in many immune system functions such as the phagocytosis and have the ability to stimulate innate immune responses, releasing inflammatory mediators [44, 79].

Vascular endothelial growth factor (VEGF), also enriched in this module, is known to play crucial roles in endothelial cell proliferation, migration, angiogenesis, vascular permeability, inhibition of apoptosis, and pathogen infection. This pathway is also involved in the interaction between pathogens and host cells as pathogens are able to encode VEGF homologs which bind to the VGFR receptor (VEGFR-2) of the host cells and then contribute to the infection process [80].

Another interesting pathway was Pattern Recognition Receptors (PRRs) which are proteins found in the host as part of the innate immune system and can also impact adaptive immunity [81]. PRRs were enriched in both positively and negatively correlated modules, however, via different genes, as detailed in Table 3. Other functions related to this module have roles in the protein modification process, cell junction assembly and substrate adhesion-dependent cell spreading. Most functions in this module are involved in infection development and stress responses.

Interestingly, an important function frequently enriched by both host and pathogen gene sets across different modules was related to the regulation of metal ions transmembrane transporters. Metal ions are essential for living organisms and play a vital role in the regulation of both microbial virulence and host immune responses. Recent studies have shown a role for metal ions in protection of the host against infections [82–85]. Therefore, it could be beneficial for the invading pathogen to express a variety of such genes in order to steal metal ions from the host.

In both groups (positively >70 and negatively-correlated <−0.7 modules), KEGG enrichment analyses of the host gene set showed similarities to previous observations during various Gram-negative infections (* Legionella *, *Salmonella *, *Shigella *, *Yersinia *, *Pertussis*, *Helicobacter pylori *).

**Fig. 6.**
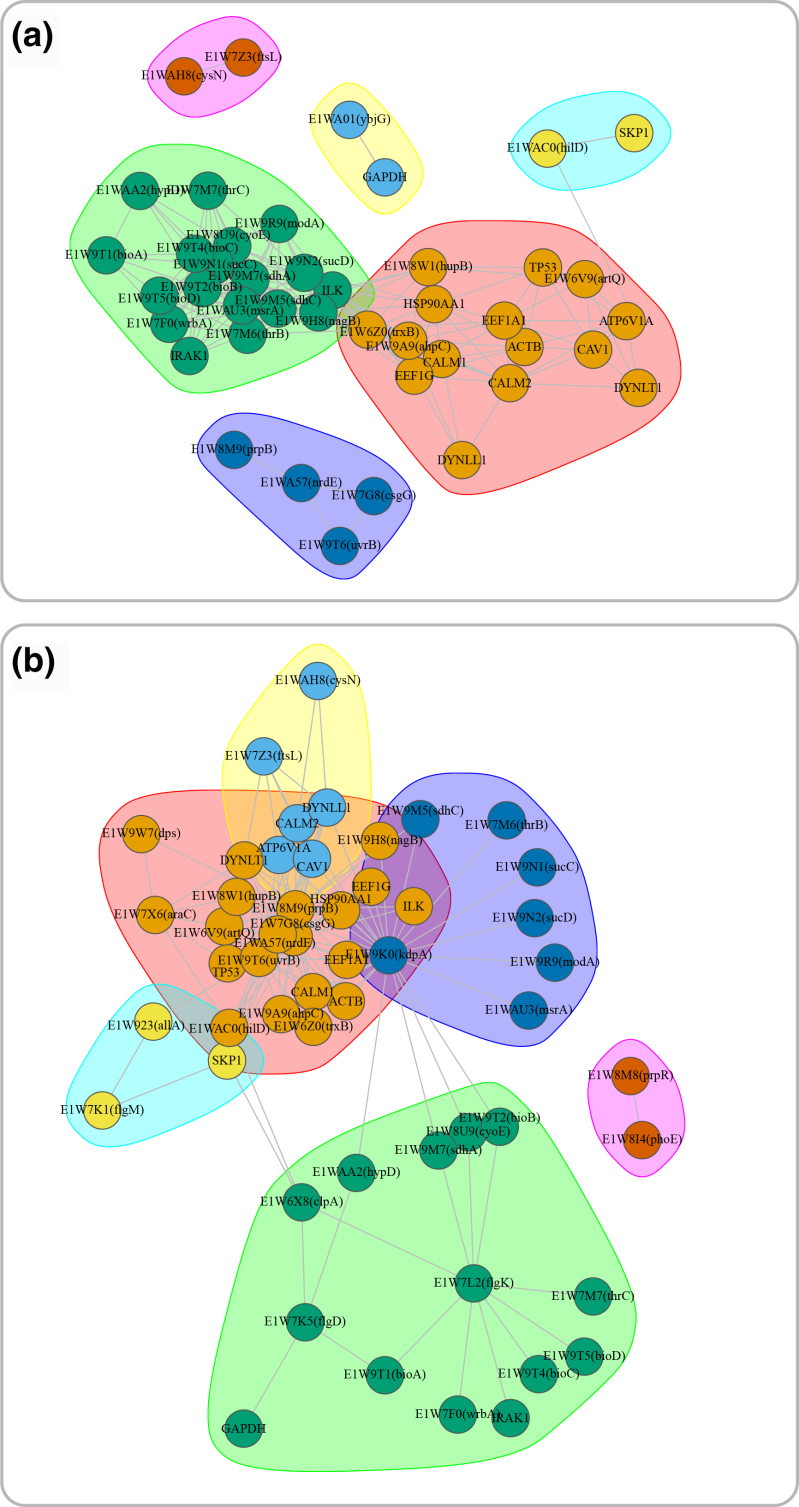
Inter- and Intra-species correlations among pathogen and host genes (Pearson correlation coefficient <−0.9 or >0.9) obtained by the ‘corrplot’ function in R resulted in 54 genes (comprising 15 host and 39 pathogen genes) which were used in the subsequent analyses to obtain co-expressed clusters.

**Fig. 7.**
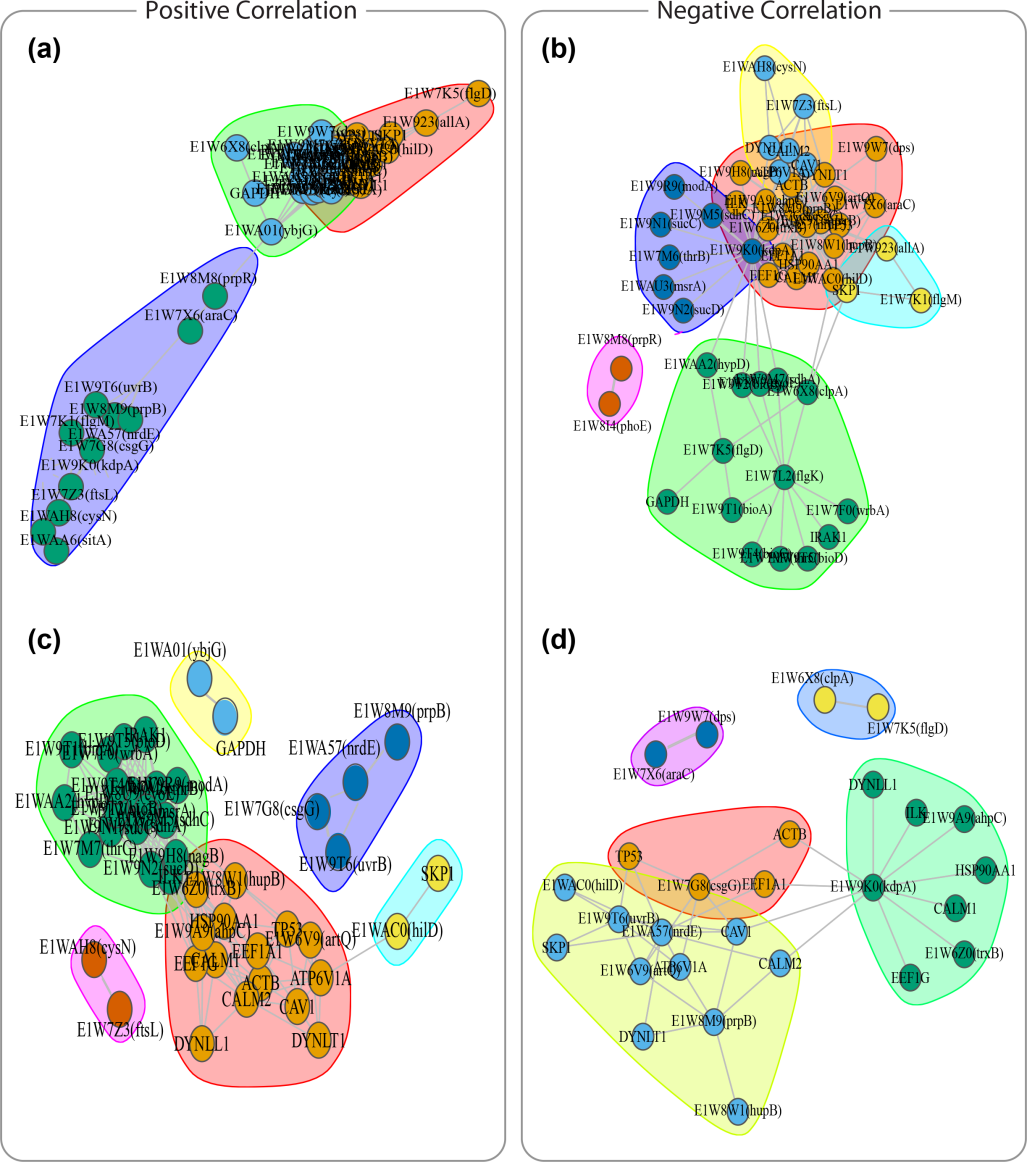
Co-expression clusters obtained by applying the Louvain algorithm on the host and pathogen correlation networks, where nodes are pathogen or host genes and edges represent correlation association (Pearson correlation coefficient less than 0.7 or 0.9 in either direction). The positively and negatively correlated modules with different cut-offs of 0.7 and 0.9 were identified, resulting in three modules in the positive correlation network, corr >0.7 (**a**); six modules in the positive correlation network with a more stringent cut-off of corr >0.9 (**b**); six modules in the negative correlation network, corr <−0.7 (**c**); five modules in the negative correlation network with a more stringent cut-off of corr <−0.7 (**d**).

Overall, we identified co-expression modules which are potentially involved in specific biological processes during early stages of the *Salmonella* infection. We then further investigated the inter-module relationships to identify genes that are key in interconnecting different modules. Accordingly, we first selected host-pathogen correlated genes to further stringent the subsequent analyses to modules whose constituent host/pathogen genes have a very similar expressional pattern during infection. This resulted in 54 genes with positive or negative correlations (Fig. 6) with corr <−0.9 or corr >0.9 forming six negative (Fig. 7a) and positive modules (Fig. 7b). We computed network-based metrics on genes within these modules and selected genes connecting different modules with high betweenness (≥40) and degree (≥10) as key inert-module connectors. The analyses uncovered the role of E1W9K0 (*kdpA*)*, CAV1*, E1WA57 (*nrdE*) and *SKP1* in interactions between four negative correlated modules (Tables S11 and S12). Additionally, E1WAC0 (*hilD*), *ATP6V1A*, *ILK*, E1W6Z0 (*trxB*), E1W8W1(*hupB*), and E1W9A9 (*ahpC*) were identified as key genes to interconnect positive correlated modules (Tables S13 and S14). Further*,* DE analyses have shown downregulation activities of *EEF1A1*, E1W8W1 (*hupB*), and *CALM2* switching off the expression of E1W6Z0 (*trxB*) in module one. Furthermore, the downregulation of *ILK* switches off the expression of E1W9H8 (*nagB*) in module three. Enrichment analyses have shown pathogen genes involved with oxidation-reduction process and potassium ion transport. Host genes mainly related to regulation of cellular response to inflammation, cellular iron ion homeostasis, and regulation of epithelial cell differentiation. Together, these observations support interactions of these genes in the early stage of infection.

## Conclusion

Here, we present the dRNASb pipeline for the network-level analyses of dual RNA-seq data. The pipeline was showcased on a *Salmonella enterica serovar* Typhimurium-infected HeLa cell model, with a detailed description of analyses performed, and results achieved. The proposed network-based analyses provide complementary information on infection processes not obtainable otherwise. In a nutshell, combining statistical analyses with network-level and functional analyses, as presented in this study, can elucidate the interplay between host and pathogen during bacterial infection and provide valuable information on which genes in the host or pathogen can be potential molecular targets for therapeutic interventions. Development of such pipelines can enhance system-level understanding of host-pathogen transcriptomic dynamics and can aid in the identification of key genes or functional interpretation of poorly annotated genes. In the future, dRNASb can be further enhanced by inclusion of machine learning models to further integrate complementary information for accurate prediction of host–pathogen interactions during the infection process.

## Supplementary Data

Supplementary material 1
